# Progressive Circuit Hyperexcitability in Mouse Neocortical Slice Cultures with Increasing Duration of Activity Silencing

**DOI:** 10.1523/ENEURO.0362-23.2024

**Published:** 2024-05-02

**Authors:** Derek L. Wise, Samuel B. Greene, Yasmin Escobedo-Lozoya, Stephen D. Van Hooser, Sacha B. Nelson

**Affiliations:** Department of Biology, Brandeis University, Waltham, Massachusetts 02454

## Abstract

Forebrain neurons deprived of activity become hyperactive when activity is restored. Rebound activity has been linked to spontaneous seizures in vivo following prolonged activity blockade. Here, we measured the time course of rebound activity and the contributing circuit mechanisms using calcium imaging, synaptic staining, and whole-cell patch clamp in organotypic slice cultures of mouse neocortex. Calcium imaging revealed hypersynchronous activity increasing in intensity with longer periods of deprivation. While activity partially recovered 3 d after slices were released from 5 d of deprivation, they were less able to recover after 10 d of deprivation. However, even after the longer period of deprivation, activity patterns eventually returned to baseline levels. The degree of deprivation-induced rebound was age-dependent, with the greatest effects occurring when silencing began in the second week. Pharmacological blockade of NMDA receptors indicated that hypersynchronous rebound activity did not require activation of Hebbian plasticity. In single-neuron recordings, input resistance roughly doubled with a concomitant increase in intrinsic excitability. Synaptic imaging of pre- and postsynaptic proteins revealed dramatic reductions in the number of presumptive synapses with a larger effect on inhibitory than excitatory synapses. Putative excitatory synapses colocalizing PSD-95 and Bassoon declined by 39 and 56% following 5 and 10 d of deprivation, but presumptive inhibitory synapses colocalizing gephyrin and VGAT declined by 55 and 73%, respectively. The results suggest that with prolonged deprivation, a progressive reduction in synapse number is accompanied by a shift in the balance between excitation and inhibition and increased cellular excitability.

## Significance Statement

When cortical activity is silenced during development, lifelong seizures often result. Here, we explored whether these seizures result from overcompensation of homeostatic recovery mechanisms. Prior work showed that neurons briefly deprived of the ability to fire action potentials compensate by becoming more excitable, increasing synaptic drive and intrinsic excitability. We found that prolonged silencing of the cortex produced a profound loss of synapse density, especially for inhibitory synapses, pointing to a circuit unable to maintain excitatory/inhibitory balance. These results show that homeostatic responses, which are normally restorative, can result in maladaptive circuit configurations when brought to extremes.

## Introduction

During development, forebrain circuits respond homeostatically to changes in activity, decreasing circuit excitability when activity increases (Kilman et al., 2002; Swanwick et al., 2006) and increasing circuit excitability to counteract decreased activity (Turrigiano et al., 1998; Desai et al., 1999). However, this normally beneficial homeostatic plasticity can be maladaptive if compensatory mechanisms overshoot normal activity levels (Nelson and Valakh, 2015). Silencing of neuronal activity during development is sufficient to produce lifelong epilepsy (Galvan et al., 2000; Lee et al., 2008): a protracted activity blockade (10–14 d) that was delivered during the second postnatal week induced a lasting susceptibility to seizures. Blocking activity later in development did not cause seizures, suggesting a particular developmental vulnerability. This developmental stage is also a time when homeostatic regulation of circuit excitability is known to be stronger than it is later in development (Desai et al., 2002).

Previous work has examined homeostatic responses to short-term (4–48 h) reductions of neuronal activity. These include changes in postsynaptic glutamate receptor trafficking (O’Brien et al., 1998; Ehlers, 2000; Gainey et al., 2009), changes in presynaptic glutamate release (Murthy et al., 2001; Frank et al., 2006; Echegoyen et al., 2007), changes in the pre- and postsynaptic strength of inhibitory synapses (Marty et al., 2000; Kilman et al., 2002; Chattopadhyaya et al., 2004; Bartley et al., 2008), and changes in cellular excitability (LeMasson et al., 1993; Desai et al., 1999).

By contrast, far less is known about the consequences of longer periods of activity blockade or about how activity recovers once it is restored. Prolonged activity deprivation increases susceptibility to seizures as described above (Galvan et al., 2000; Lee et al., 2008), but the underlying mechanisms are not known. Persistent hyperactivity following the removal of activity blockade could reflect altered cellular excitability and/or changes in the strength and number of excitatory and inhibitory synapses brought about as part of the initial homeostatic response. In addition, other forms of plasticity, such as Hebbian long-term potentiation (LTP) or depression (LTD) could further alter synaptic strength once network activity resumes.

To learn more about the kinetics of this hypersynchronous activity overshoot, we blocked neuronal firing with the sodium channel blocker tetrodotoxin (TTX) in organotypic cortical slice cultures and then monitored network activity with calcium imaging at various times after removal of TTX. To assess the impact of Hebbian plasticity, we compared recovery in the presence and absence of the NMDA receptor blocker APV. To begin to tease apart some of the cellular and synaptic factors that contribute to abnormal network activity following protracted activity blockade, we then measured cellular excitability in whole-cell recordings and imaged presumptive excitatory and inhibitory synapses with super-resolution confocal microscopy. The results reveal complex progressive and multifactorial changes in these cortical networks that likely contribute to their abnormal hypersynchronous activity and that may contribute to seizures following activity blockade in vivo.

## Materials and Methods

### Organotypic cortical slice culture

Slice cultures were prepared from 7-d-old wild-type C57-B6J mice, of both sexes, anesthetized with 40 μl of a standard mixture containing ketamine (20 mg/ml), xylazine (2.5 mg/ml), and acepromazine (0.5 mg/ml) (Stoppini et al., 1991). After being embedded in agarose within a cylindrical chamber that slightly narrows at its opening, the brain was cut using a compresstome (Precisionary Instruments) in filtered ice-chilled artificial cerebrospinal fluid (ACSF; containing 126 mM NaCl, 25 mM NaHCO_3_, 3 mM KCl, 1 mM NaH_2_PO_4_ H_2_O, 25 mM dextrose, 2 mM CaCl, and 2 mM MgCl_2_, with an osmolarity of 315–319 mOsm) to achieve a 300 μm thickness. The brain slices were grown on Millipore Millicell inserts (MilliporeSigma) in six-well dishes containing SCM media [1× MEM (MilliporeSigma), 1× GlutaMAX (Invitrogen, Thermo Fisher Scientific), 1 mM CaCl_2_, 2 mM MgSO_4_, 12.9 mM dextrose, 0.08% ascorbic acid, 18 mM NaHCO_3_, 35 mM HEPES (pH 7.5), 1 μg/ml insulin, and 20% horse serum (heat inactivated, MilliporeSigma), at pH 7.45 and with an osmolarity of 305 mOsm]. An antibiotic mixture of 1,000 units/ml Pen-Strep (Invitrogen, Thermo Fisher Scientific) and 50 μg/ml gentamicin (MilliporeSigma) was applied for the first 24 h in a 35°C incubator with 5% CO_2_ in the atmosphere. After changing into nonantibiotic media, media changes were performed every 2 d for the course of experiments. One day after dissection, 1 μl AAV9/hSyn/GCaMP6f virus (Addgene) diluted 1–5× from commercial titer (2.8 × 10^13^ to ∼1 × 10^12^ units/ml) in ACSF, was applied to the surface of the slice just inferior to the cortex.

Cultures were kept active for 15 d in total for most of our experiments, starting at equivalent postnatal day (EP) 7 and stretching to EP25. For one experiment, experiments continued until EP32. During the first 5 d in vitro (DIV), cultures recovered from dissection. Tetrodotoxin (TTX) was dissolved in warmed media at 500 nM concentration and applied to some cultures during a media change beginning at 5 or 10 DIV for most experiments. Monitoring of calcium transients in several cultures confirmed that full silencing of these cultures occurred within 5–10 min after TTX application and that the drug was still effective at suppressing calcium activity after 48 h. Recovery experiments took place following a triplicate media change into fresh nondrug media and 3 d of time to allow for cultures to equilibrate.

All animal procedures were performed in accordance with the regulations of the Animal Care Committee of Brandeis University.

### Calcium imaging

Layer 5 of the somatosensory cortex was imaged at 33 frames per second for 10 min on a Leica DMI 6000B microscope (Leica Microsystems) with a Yokogawa CSU W1 confocal spinning disc unit, using an Andor Neo sCMOS camera operated by Andor IQ3 (Andor Technology) under perfusion with 35–37°C oxygenated ACSF (the same solution as for dissection). Around half a millimeter square was imaged with a 10× objective (Carl Zeiss), visualizing many hundreds of cells. The recordings of slices that had been treated with TTX were conducted in ACSF that was free of drug, allowing for cellular activity.

### Calcium analysis

Videos were processed in a custom MATLAB suite (Li et al., 2008; RRID:SCR_023369). Ellipsoidal cellular ROIs of neuron somata were selected by hand using a standard deviation projection of the videos, and then their average intensity was calculated at each frame and recorded as a raw time trace. These traces were normalized by a standard Δ*F*/*F* formula, (value − baseline) / baseline, where the baseline was defined as the average of timepoints where a cell had a low coefficient of variation for the following 50 frames. Noisy or especially dim data was discarded, both for individual cells and also for slices that had <15 cells that fit our criteria.

Periods of activity, termed “up states” (Cossart et al., 2003; Johnson and Buonomano, 2007), were detected with knowledge of the slope of the mean trace of all cells’ activity. Positive deflections in slope > 0.15 Δ*F*/*F* in the calcium activity were taken to indicate firing. The threshold was experimentally determined to separate subthreshold events and noise (uncorrelated between cells) from firing, which was often correlated across neurons. Up state boundaries were defined by a pause in firing exceeding a user-defined refractory period, generally between 30 and 120 frames (1–4 s).

Up states were counted and then characterized in several ways. The correlation coefficient between each cell pair was determined during each up state and averaged across all cell pairs and all up states (synchrony). We also measured the correlation coefficient between the activity of a cell in one up state to its activity in each other up state (stereotypy), using the first 3 s of each firing period. The duration was defined as the time between the first and last frame when a significant positive slope was observed, meaning that the period at the end of each up state during which the calcium signal falls exponentially was not considered to be a part of the up state.

We did not attempt to quantify up state amplitudes using calcium imaging because this slow modality provides poor information about the number of action potentials occurring within a short synchronous burst since many factors, such as GCaMP6f expression levels, contribute to overall amplitude differences across experiments and between cells within an experiment.

### Electrophysiology

Intrinsic excitability was measured from current-clamp recordings of visually identified Layer 5 pyramidal cells in the primary somatosensory cortex in response to a series of 1-s-long current pulses from a resting membrane potential of −70 mV. Slice cultures were taken from the incubator directly to the electrophysiology rig on a cutout section of insert and allowed to rest for 20 min at 35–37°C under oxygenated ACSF (the same as from dissection) before the recording began. Glass recording pipettes (3–5 MΩ resistance) were filled with internal solution [containing (in mM) 100 K-gluconate, 20 KCl, 10 HEPES, 4 Mg-ATP, 0.3 Na-GTP, 10 Na-phosphocreatine, and 0.05% biocytin]. Recordings were made using an Axopatch 200B amplifier (Axon Instruments), filtered at 10 kHz without correction for liquid junction potentials, and digitized at 20 kHz using a National Instruments data acquisition board in a Dell computer using custom Igor Pro software (WaveMetrics). Further analysis was performed in Igor, MATLAB (MathWorks), or R.

### Synaptic imaging

Slice cultures were fixed with 3% glyoxal for 30 min at 4°C and then for 30 min at room temperature (Richter et al., 2018). Fixed slices were quenched in ammonium chloride/glycine solution (10 mM each) for 5 min, washed three times into PBS, and stored at 4°C until staining in PBS containing 1 μM sodium azide.

For staining, single-hemisphere slices were removed from culturing membranes and first exposed to CUBIC-1 solution overnight at 37°C on a shaker (Susaki et al., 2015). These slices were then treated with the DeepLabel staining system (Logos Biosystems) suitable for thick samples, using a version of the DeepLabel protocol featuring overnight washes in each proprietary solution. Primary antibodies, for our excitatory synaptic stain, were anti-PSD-95 (mouse, Synaptic Systems, 124-011), and anti-Bassoon (rabbit, Synaptic Systems, 141-003). Secondary antibodies were goat anti-mouse Alexa Fluor Plus 555 (Invitrogen, A32727) and goat anti-rabbit Alexa Fluor Plus 488 (Invitrogen, A32732). All antibodies here were diluted 1:2,000 for use. In the final wash, DAPI (Thermo Fisher Scientific, 62248) was added at 1 μg/ml (1:1,000) for 20 min at 37°C on a shaker to stain nuclei. Slices were carefully mounted with a proprietary solution, DeepLabel XClarity, and allowed to set overnight before imaging.

Inhibitory synapses were stained using anti-VGAT (chicken, Synaptic Systems, 131-006) and anti-gephyrin (mouse, Synaptic Systems, 147-111). Secondary antibodies consisted of goat anti-chicken Alexa Fluor Plus 647 (Invitrogen, A32933) and goat anti-mouse Alexa Fluor Plus 488 (Invitrogen, A32723) with DAPI included as above. In addition, the 1:2,000 dilution for these antibodies obtained good staining.

Imaging of these samples was performed on the Zeiss AiryScan confocal microscope (Carl Zeiss) at 63×. Imaging was confined to Layer 5 of the somatosensory cortex. We took *z*-stacks of 16 frames 0.2 μm apart, covering a total thickness of 3 μm. With X/Y dimensions of 72 × 72 μm, the total volume surveyed for these experiments was 15,552 μm^3^.

### Synaptic imaging analysis

Raw images were passed through the AiryScan image processing system native to Zeiss Zen Black, increasing their resolution by roughly twofold. Using FIJI (Schindelin et al., 2012) and MATLAB, these images were then converted into single-channel .TIFF files, scaled to normalize for intensity over depth, and deconvoluted with a point-spread-function inferred from TetraSpeck 100 nm fluorescent microspheres (Thermo Fisher Scientific T7279) imaged with the same optical setup and processing. To identify synaptic puncta in our channels of interest, we used a system of thresholding based on an estimate of the likely maximal brightness of noise in our image. A skewed normal distribution was fitted to the histogram of pixel intensity values, and the percent likelihood that a given intensity value belonged to this distribution was computed. The inverse of this, the likelihood that a value was part of the signal, was transformed into an intensity threshold for peak detection. An experimenter blinded to the condition settled on a threshold for each stack that excluded objects that were clearly out of focus. Any dimmer object or part of an object was not considered a potential region of interest (ROI).

Thresholded ROIs had edges defined by a second, slightly lower, threshold (Wise et al., 2023) and then dilated by three pixels to ensure counting pixels close to the threshold. ROIs were resegmented using a watershed algorithm into component objects and then restricted to the pixels, which were within 30% of the difference between the puncta's peak and the local background. ROIs that were thereafter under eight pixels (0.013 μm^3^) in volume were excluded from the final dataset. Finally, the colocalization of each punctum with its synaptic partner was assessed. Puncta were considered colocalized if they were within two pixels of a potential partner. These colocalized puncta are the population reported here for their properties—density and volume.

Cell counts of a 461 × 461 μm region containing Layer 5 of the somatosensory cortex were performed at 10× for the full extent in *z* using CellProfiler (Stirling et al., 2021) to segment and count nuclei. Density was then calculated for one stack per imaged slice.

### Statistics

Unless otherwise stated, grouped data were analyzed by one- or two-way ANOVA followed by post hoc testing (Tukey's honest significant difference test). Error bars indicate standard error of the mean.

### Code accessibility

Novel MATLAB code for PC/Mac detailing our calcium analyses is available online in a GitHub directory, RRID:SCR_023369.

## Results

### Calcium transients demonstrate a progressive loss of activity homeostasis with prolonged deprivation

**Figure 1.**
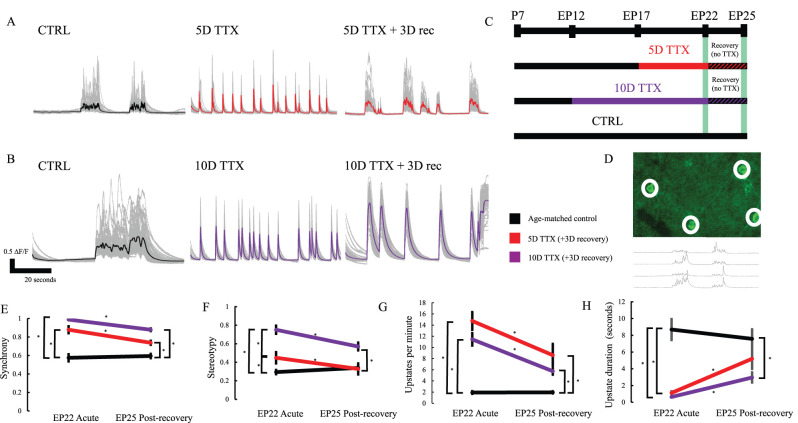
Population activity in organotypic cortical slice cultures is hyperactive and hypersynchronous upon release from prolonged activity deprivation and following 3 d recovery. ***A***, Traces (mean in color, individual cells in gray) show calcium transients recorded from individual cells after normal activity (CTRL, black, left), after release from 5D of TTX (center, red), or after recovery for 3D in normal media before imaging (right, red). ***B***, Same as ***A*** but following 10D of TTX exposure (purple). ***C***, Experimental timeline (*n* = 23 EP22 CTRL, 27 EP25 CTRL, 16 EP22 5D TTX, 19 EP25 TTX 3D recovery, 14 EP22 10D TTX, and 12 EP25 10D TTX 3D recovery slices). ***D***, Representative single-frame CTRL calcium imaging with white circles representing cellular ROIs with high GCAMP6f fluorescence. Traces below show these four cells over 1 min of imaging. ***E***, Synchrony, averaged across all pairs and up states. Black, age-matched control; red, 5D of TTX; purple, 10D of TTX, immediately after activity deprivation (EP22 acute) and following an additional 3D of recovery in normal media (EP25 postrecovery). Stars: *p* < 0.05 (two-way ANOVA, Tukey's post hoc test). ***F***, Stereotypy, calculated as autocorrelation at zero lag of each cell with its activity across up states, averaged across all cells. Significance stars as in ***E***. ***G***, Frequency of activity, as up states per minute. Significance stars as in ***E***. ***H***, Average up state duration, across all up states in the 10 min recording window, in seconds. Significance stars as in ***E***. Extended Data Figure 1-1 explores whether widespread cell death results from prolonged TTX silencing.

Spontaneous activity in cortical organotypic slice cultures becomes progressively more complex and frequent over the first few weeks in vitro (Johnson and Buonomano, 2007), characterized by periodic population firing events, termed “up states,” in which neurons depolarize to make activity propagation more likely (Cossart et al., 2003). These up states were evident in GCaMP6f fluorescence recordings of network activity, monitored at 15–18 d in vitro (EP 22–25; Fig. 1*A,B*, left). The calcium imaging approach used had neither the temporal resolution nor the precise calibration to be able to directly infer action potential firing rates from calcium transients. Four different measures were used to quantify network activity: synchrony was quantified as between-cell correlation at zero lag between-cell pairs within the period of each up state and then averaged across all cells and all up states; stereotypy was the within-cell correlation of a cell's activity in one up state with its activity in other up states, calculated per cell on each pair of up states then averaged across all up state pairs and all cells; up state frequency was simply the count of up states within an average minute; up state duration was assessed as the time between the first period of increasing slope in an up state and the last. Activity in healthy slices was characterized by several consistent features: (1) a modest frequency of network up states (mean, ∼1.8 up states per minute) remaining relatively constant from EP22 and EP25; (2) calcium transients in simultaneously imaged cells, although coarsely synchronous in that they were grouped into 5- to 30-s-long up states (mean, 8.7 s), were not tightly synchronized at more rapid time scales (synchrony measures below 0.6, Fig. 1*E*); and (3) activity in simultaneously recorded cells often occurred in a different order in different up states (low levels of stereotypy, Fig. 1*F*), with many cells active in some up states and not others. This suggests a circuit that has achieved a relatively balanced activity state in culture following the regrowth of severed connections from the dissection process.

10.1523/ENEURO.0362-23.2024.f1-1 Figure 1-1 **No significant cell death occurs with prolonged tetrodotoxin exposure. A.** Example 10x image from Zeiss Airyscan of DAPI-stained nuclei (blue) in layer 5 of somatosensory cortex in 461 x 461 μm area. Full z extent was imaged for each stack, so the total volume differed in for each sample. **B.** CellProfiler calls (red outline) of good nuclei with inset showing examples of rejected blurred nuclei out of focus (purple outlines) and accepted crisp outlines of counted nuclei (green outlines). **C.** Bar graph of total nuclei count in our three conditions, from the same slices used in synaptic imaging analysis. 1-way ANOVA and posthoc Tukey’s both p > 0.05. **D.** Examples of accepted cell density (red outlines) from all three conditions. Download Figure 1-1, TIF file.

Following prolonged activity blockade, organotypic cultures exhibited a highly stereotyped and synchronized state of epileptiform activity (Fig. 1*A,B*, middle) as previously described for slice culture and dissociated cultures (Ramakers et al., 1990; Trasande and Ramirez, 2007; Koch et al., 2010). Up states were observed significantly more frequently after silencing, shifting from a mean of 1.9 events/min in age-matched control to14.7 events/min at 5D TTX to 11.5 events/min with 10D TTX. When one neuron fired, most or all other neighbors rapidly became active as well, leading to a synchrony approaching a perfect correlation of 1 (mean, 0.88 5D TTX, 0.99 10D TTX). Each of the up states involved a highly stereotyped burst, in which each up state appeared as a sudden, brief flash (stereotypy mean, 0.45 5D TTX, 0.75 10D TTX; an increase of 55 and 158% respective relative to control activity). The up state duration dropped from a mean of 8.7 s in control activity to 1.1 s following 5D TTX and to 0.6 s following 10D TTX. This highly synchronous rebound firing after activity deprivation occurred in every slice examined, likely reflecting the persisting homeostatic drive accumulating in the absence of feedback during TTX silencing. The greater increase in synchrony and stereotypy after 10D TTX suggests that homeostatic changes continue to occur even after the initial 5D of deprivation.

One exception to this pattern was that up state frequency was increased more following 5D of TTX than 10D of TTX, but this difference was not significant (5D vs 10D TTX acute *p* = 0.26 Tukey's post hoc test, 5D vs 10D TTX). The duration of up states was also not significantly different immediately after 5 and 10D TTX (*p* = 0.99). In both cases, post-TTX activity occurs in short synchronous bursts. If, however, the 10D TTX bursts had higher overall firing rates, this may have led to more rapid termination of each burst, through greater recruitment of inhibition, synaptic depression, and accommodation.

The greater hyperexcitability seen after 10D TTX might also be associated with a slower or less complete recovery following the period of deprivation. To assess this, we monitored activity following 3 d in normal ACSF. Each of the three metrics examined showed some degree of recovery, but most did not recover fully after 3 d, and in general, recovery was more complete following 5D TTX than following 10D TTX. For example, after 5D TTX, synchrony recovered to an average synchrony of 0.74, approximately halfway from the acute value of 0.88 after 10D TTX to the control value of 0.59, but this remained significantly different from control (*p* < 0.01 Tukey's post hoc test). After 10D TTX, synchrony recovered to an average of 0.88, which was only about a quarter of the way from the acute value of 0.99 to the baseline value. This synchrony after recovery was significantly different from baseline (*p* < 0.01) as well as from the recovery after 5D TTX (*p* = 0.01). This differential recovery was even more striking for stereotypy, which recovered completely following 5D TTX (mean, 0.33 vs 0.34 CTRL, *p* = 0.99) but only partially following 10D TTX (mean, 0.57 vs 0.34 CTRL, *p* < 0.01). Up state duration also showed more complete recovery following 5D TTX compared with 10D TTX (CTRL mean, 7.6 s; 5D TTX recovery, 5.2 s; and 10D TTX recovery, 3.0 s). The 5D TTX recovery was not significantly different from the control (*p* = 0.43), but the 10D recovery was still significantly different from CTRL (*p* = 0.01). Similar to the acute effects, the exception to this pattern was the up state frequency where both after 5D TTX and after 10D TTX the recovery was incomplete (*p* < 0.01 and *p* = 0.03 vs CTRL respectively). Qualitatively, up states after recovery from 5D TTX sometimes resembled the control state (Fig. 1*A*), although this was not always the case. In contrast, 3D recovery was usually insufficient after 10D TTX to restore a more asynchronous pattern of network activity.

It is possible that, since our recovery analysis compared cultures that had 10D of TTX beginning at EP12 to cultures that had 5D of TTX beginning at EP17, the earlier initiation of prolonged silencing was a better explanation than the increased duration for why the cultures remain persistently disrupted. However, we also tested recovery after 5D TTX when deprivation began at EP12. While the initial rebound hyperactivity from 5D TTX at this earlier age is somewhat more severe (*n* = 17 EP17, *n* = 14 EP22; synchrony mean 0.94 at EP17, 0.87 at EP22; stereotypy mean 0.66 at EP17, 0.44 at EP22), there was similar recovery after 3D of recovery at the two ages. EP20 3D recovery showed 0.74 mean synchrony, compared with EP25 3D recovery at 0.78 mean synchrony (*n* = 10 EP20, *n* = 14 P25; two-tailed *T*-test *p* = 0.40); 0.40 stereotypy mean for EP20, and 0.33 mean for EP25 (two-tailed *t*-test *p* = 0.22). Repeating the two-way ANOVA to compare these effects to 10D TTX and to condition-matched CTRL resulted in similar conclusions to those obtained from the measurements made at EP25. With regard to synchrony, 5D TTX starting EP12 was still statistically significantly different from CTRL (Tukey's *p* < 0.01), but the somewhat higher value was intermediate to but now not significantly different from 10D TTX 3D recovery starting at EP12 (*p* = 0.41). Stereotypy, as above, reveals a full recovery at 3D following 5D TTX starting EP12 (*p* = 0.85 vs CTRL) but is now not significantly more recovered than 10D TTX 3D recovery starting at EP12 (*p* = 0.14). We conclude that 5D TTX at this earlier time point is insufficient to yield lasting hyperactivity and still produces an intermediate state to 10D TTX starting at the same time.

Overall, calcium imaging reveals that populations of neurons transiently overshoot their homeostatic set point when deprived of activity for a prolonged period and this hyperexcitability is more extreme following a longer period of deprivation. A 3 d period of recovery in normal media results in an incomplete recovery that is correspondingly less restorative when the deprivation is more prolonged.

### Longer periods of recovery produce a more complete return to baseline activity

**Figure 2.**
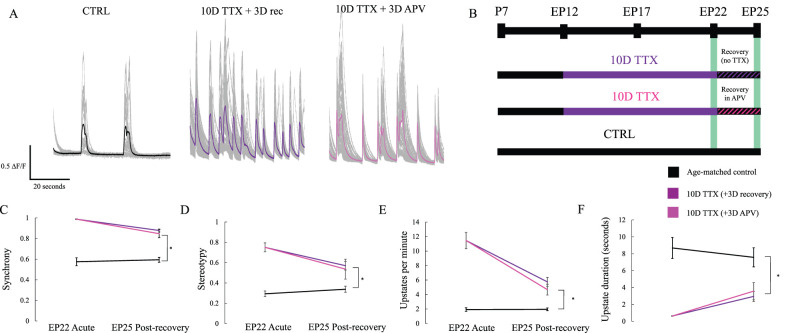
Longer recovery following prolonged deprivation does eventually equilibrate in healthy activity patterns. ***A***, Traces (mean in purple, individual cells in gray) show calcium transients recorded from individual cells after normal activity (10D TTX, left; same group as in Fig. 1), after 3D of recovery in normal media following 10D of TTX (center, as in Fig. 1), after 7D recovery (*n* = 7 for EP29 age-matched CTRL slices, 10 10D TTX 7D recovery, not shown), and after recovering for 10D in total (right, *n* = 10 for EP32 age-matched CTRL slices, 16 10D TTX 10D recovery). ***B***, Experimental timeline. ***C***, Synchrony progressively recovers by 7D, fully recovered by 10D. Stars: *p* < 0.05 (two-way ANOVA, Tukey's post hoc test). ***D***, Stereotypy, otherwise as in ***C***. ***E***, Up state frequency per minute, otherwise as in ***C***. ***F***, Up state average duration in seconds, otherwise as in ***C***.

Because 3D was clearly insufficient, we investigated whether longer recovery could better ameliorate the lasting impact of silencing (Fig. 2). Specifically, we extended the recovery period after 10D of TTX to 7 or 10D. For synchrony (P32 CTRL vs 10D TTX 10D recovery, Tukey's *p* = 0.56), stereotypy (*p* = 0.37), up state frequency (*p* = 0.99), and duration (*p* = 0.81), there was complete recovery back down to age-matched control values. Critically, our results do not imply that recovered circuits are in all ways identical to their original state but only that our population activity metrics capturing average events grossly recover to baseline level. A circuit of this type could arrive at the same overall balance of activity but still be more fragile to additional insult or strong stimulus.

### Hyperexcitability is most pronounced when activity blockade is initiated prior to the end of the second postnatal week

**Figure 3.**
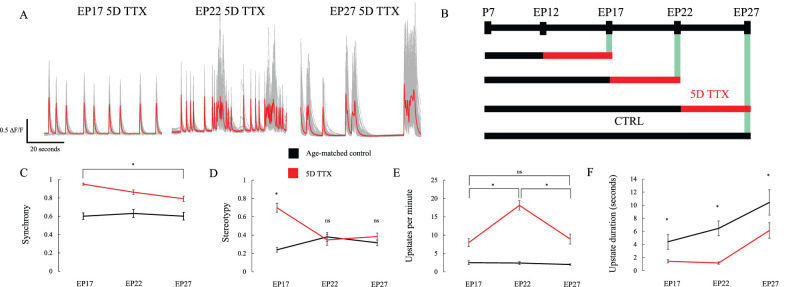
The overshoot following 5D of TTX is age-dependent, with the strongest effects occurring when initiated prior to the end of the second postnatal week. ***A***, Traces (mean in color, individual cells in gray) show calcium transients recorded from individual cells after 5D of TTX (ending EP17, left, *n* = 15 CTRL and 18 5D TTX slices; EP22, middle, *n* = 15 CTRL and 16 5D TTX slices; EP27, late, *n* = 23 CTRL and 26 5D TTX slices). ***B***, Experimental timeline. ***C***, Synchrony overshoot is strongest at EP17 and is significantly reduced by EP27. Stars: *p* < 0.05 (two-way ANOVA, Tukey's post hoc test). ***D***, Stereotypy recovers back to baseline conditions by EP22, significance stars as in ***C***. ***E***, Up state frequency displays a nonmonotonic relationship, significance stars as in ***C***. ***F***, Up state duration reduced following silencing, significance stars as in ***C***.

Homeostatic plasticity is regulated developmentally, with stronger plasticity observed earlier in development (Desai et al., 2002). The maladaptive response to activity deprivation in vivo is also strongly regulated by development and does not lead to recurring seizures if initiated after the end of the second postnatal week of development (Galvan et al., 2000; Lee et al., 2008). To see if this correlated with the developmental sensitivity to epileptiform activity in slice culture, we initiated 5 d of activity deprivation beginning at progressively later equivalent ages from EP12 to EP22 and measured the resulting hyperactivity at EP17, EP22, and EP27 (Fig. 3). Synchrony (Fig. 3*C*) and stereotypy (Fig. 3*D*) indices revealed greater hyperactivity for deprivation begun earlier. Synchrony had significantly less effect by EP27 with 5D TTX when compared with EP17 (*p* < 0.01 Tukey's post hoc test), whereas stereotypy was actually no longer statistically different from age-matched control by EP22 TTX (from *p* < 0.01 at EP17 to *p* = 0.99 at EP22 and *p* = 0.78 at EP27). For peak frequency, the effect of development was nonmonotonic, with the largest effect evident at the intermediate age (Fig. 3*E*). Duration of up state events remains briefer following deprivation, no matter what age silencing begins (*p* < 0.01 at all ages, Fig. 3*F*). This may reflect an interaction between progressively enhanced activity which increases the number of firing events and progressively lengthened intervals between high activity bursts, which tends to reduce this measure.

### Pharmacological block of NMDA receptors suggests Hebbian mechanisms make little contribution to circuit hyperexcitability

**Figure 4.**
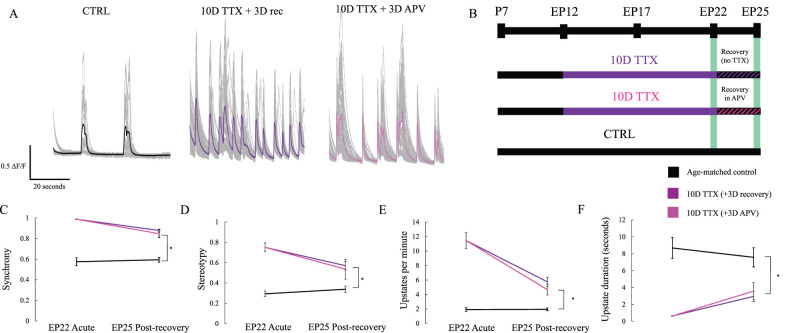
Maladaptive plasticity does not require activation of NMDA receptors during the recovery period. ***A***, Traces (mean in color, individual cells in gray) show calcium transients recorded from individual cells after normal activity (CTRL, black, left; samples from Fig. 1), after 3D of recovery in normal media following 10D of TTX (center, purple, from Fig. 1), or after recovering in 3D APV before imaging (right, pink; *n* = 10 10D TTX 3D APV slices). ***B***, Experimental timeline. ***C***, Synchrony remains significantly elevated after 3D, but this is not significantly altered by the presence or absence of APV during the recovery period. Stars: *p* < 0.05 (two-way ANOVA, Tukey's post hoc test). ***D***, Stereotypy, otherwise as in ***C***. ***E***, Up state frequency per minute, otherwise as in ***C***. ***F***, Up state average duration, otherwise as in ***C***.

Synaptic scaling at neocortical synapses is known not to require activation of NMDA receptors because blockade of NMDA receptors does not itself produce scaling of AMPA-mediated transmission (Turrigiano et al., 1998). However, following release from activity blockade, elevated network activity might lead to enhanced NMDA-dependent Hebbian plasticity, thus “locking in” the initial homeostatic increase in synaptic strength. To test for this possibility, we compared activity in normal ACSF following either 3 d of recovery with NMDA receptors blocked with 2-amino-5-phosphonopentanoic acid (APV) or with NMDA receptors available for normal activation. The concentration of APV used (50 µM) is known to be sufficient to block long-term potentiation (LTP) at neocortical synapses (Crair and Malenka, 1995; Sjöström et al., 2001). We observed comparable recovery across each of our three metrics regardless of whether NMDA receptors were blocked or not (Fig. 4, 10D TTX 3D APV vs 10D TTX 3D recovery synchrony *p* = 0.99, stereotypy *p* = 0.98, up state frequency *p* = 0.87, duration *p* = 0.99). In both cases, slices failed to recover back to age-matched baseline (10D TTX 3D APV vs P25 CTRL synchrony *p* < 0.01, stereotypy *p* = 0.04, up state frequency *p* = 0.04) although the duration of up states is somewhat statistically closer to baseline following recovery in APV than without APV due to variance (*p* = 0.19). Since recovery was not enhanced by blocking NMDA receptors, we conclude that continued network hyperactivity after 3 d of recovery is a persistent consequence of homeostatic plasticity, rather than a consequence of Hebbian plasticity engaged during the subsequent period of highly synchronous activity.

### Intrinsic excitability of L5 pyramidal neurons is slightly increased, and input resistance is increased twofold, with prolonged deprivation

**Figure 5.**
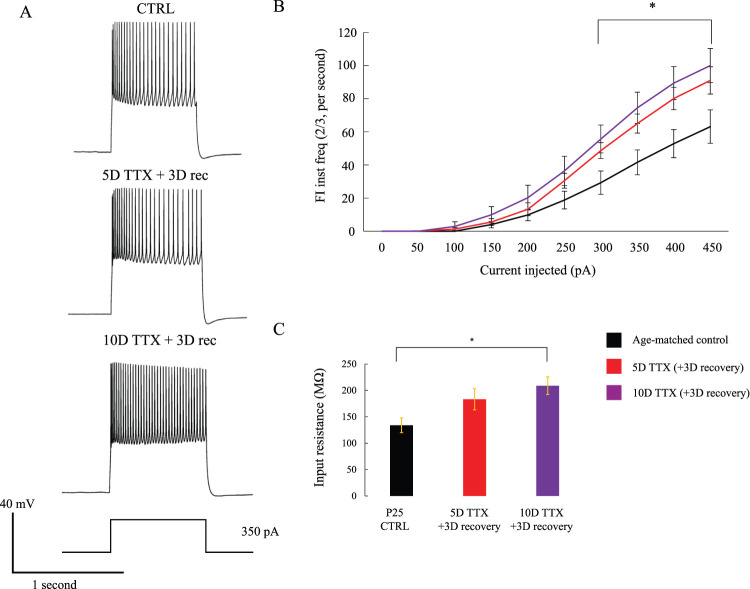
Intrinsic excitability measurements from L5 pyramidal neurons reveal an increase in instantaneous firing rate and a large increase in input resistance, with no persistent change in rheobase. ***A***, Single-trial traces of current-clamped neurons in each condition most closely approximating the mean firing observed in each condition (EP25 age-matched control, *n* = 14 cells; EP25 5D TTX 3D recovery, *n* = 12 cells; and EP25 10D TTX 3D recovery, *n* = 15 cells) with 350 pA stimulation. ***B***, Instantaneous firing rate over current injection steps from 0 to 450 pA (two-way repeated measures ANOVA, *p* < 0.05 between-subject effect of condition, within-subject effect of current step, and interaction of group and current). The star represents the significance between 10D TTX and age-matched control following this ANOVA and Tukey's honestly significant difference test (*p* < 0.05). ***C***, Input resistance from recorded cells, averaged across trials. The star represents significance following one-way ANOVA and Tukey's post hoc *p* < 0.05.

To begin to reveal which cellular and synaptic mechanisms of homeostatic plasticity might contribute to persistent hyperexcitability, we examined the intrinsic excitability of pyramidal neurons after a 3 d recovery period following TTX silencing for 5 or 10 d. Specifically, we measured frequency–current (FI) curves over a range of 0–450 pA during whole-cell current-clamp recordings (Fig. 5). Many of our 10D TTX 3D recovery cells inactivated after a few action potentials at higher current steps, so we relied instead on instantaneous firing rate between the second and third spike (FI ⅔, Fig. 5*B*). Other measures of firing we calculated included the interval between the first and second spike and the average number of action potentials (excluding periods of inactivating firing), yielding similar conclusions that were noisier (not shown; FI 1/2 and standard FI). The instantaneous FI from spikes 2–3 showed a main effect for group and current level, as well as a significant interaction effect of group and current levels (two-way repeated measures ANOVA, *F* = 3.0, *p* = 0.04 for between-subject effect of condition, *F* = 173.3, *p* < 0.01 for within-subject effect of current step, *F* = 2.8, *p* = 0.02 for interaction of group and current). Post hoc tests revealed an increase in firing from CTRL to 10D TTX 3D recovery at 300, 350, 400, and 450 pA (Tukey's; *p* = 0.03, *p* = 0.01, *p* = 0.01, *p* = 0.02, respectively), although 5D TTX was never significantly different than CTRL (closest was *p* = 0.10 at 400 pA). We made some attempts to quantify FI behavior in neurons acutely withdrawn from TTX, but 10D of TTX consistently produced cells that inactivated even after a single spike, making quantitative comparisons difficult.

In addition to greater suprathreshold firing, deprivation made neurons more excitable by increasing input resistance at rest. There was a progressive increase in input resistance with increasing deprivation duration (Fig. 5*C*, one-way ANOVA, *F* = 5.23, *p* = 0.01) that roughly doubled and was significantly different from age-matched control by 10D of TTX (CTRL mean, 133.8 MΩ; 5D TTX 3D recovery, 183.2; 10D TTX 3D recovery, 208.9; *p* = 0.13 for CTRL vs 5D TTX 3D recovery, *p* = 0.01 for CTRL vs 10D TTX 3D recovery). Increased input resistance leads to greater firing because a given level of current injection produces stronger depolarization. We calculated the extrapolated intercept of the FI curve as the rheobase, the threshold current for a single spike, and found it to not significantly differ across conditions (one-way ANOVA, *F* = 0.75, *p* = 0.48). It may be that a small difference, <50 pA, does exist but that our FI step size was too crude to reveal it. It appears that the increase in input resistance may have contributed to the increase in instantaneous firing we see.

Even given 3 d of recovery, it is clear that intrinsic excitability in our cultures did not fully recover. The shift in F/I dynamics was similar following 5 or 10 d of TTX. However, input resistance underwent a progressive change with increasing silencing duration.

### A decrease in excitatory synapse density, but a larger decrease in inhibitory synapse density, indicate a shift in E/I balance

To measure the impact of progressive deprivation and recovery on cortical synapses, we visualized synapse density and size using super-resolution microscopy (Zeiss AiryScan; see Materials and Methods). Putative excitatory synapses were defined by the colocalization of presynaptic puncta (Bassoon) with postsynaptic puncta (excitatory-specific PSD-95). This method has recently been calibrated and compared with electron microscopy in an analogous preparation (Wise et al., 2023).

**Figure 6.**
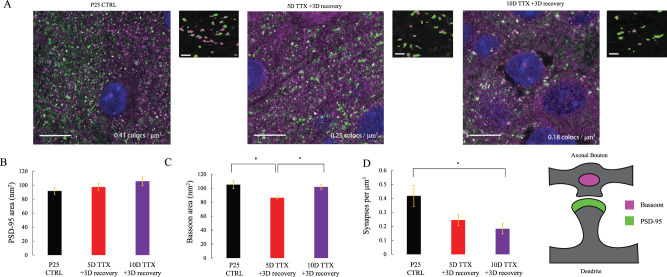
Excitatory synapse number progressively declines after 10D of TTX activity deprivation. ***A***, Representative example images, each a single *z*-frame, of organotypic slice culture tissue stained with immunofluorescent markers for the postsynapse (PSD-95, green) and presynapse (Bassoon, magenta), schematized on the far right. The scale bar on the large image is 10 μm. The right panel of each duo shows a zoomed-in (100 pixel × 100 pixel, or 72 μm square) view of a single area with thresholded colocalized puncta pseudocolored and a background of the Bassoon stain in greyscale. The scale bar on the small image is 1 μm. *n* = 7 EP25 CTRL slices, 11 EP25 5D TTX 3D recovery, 8 EP2510D TTX 3D recovery. ***B***, Average 2D cross-sectional area (in frame with the brightest pixel) of PSD-95 puncta colocalized with synaptic partners computed over a 16.6 mm^3^ area. Compared are EP25 age-matched untreated slices with 5D TTX + 3D recovery and 10D TTX + 3D recovery slices. Means are 91.8 nm^2^ (CTRL), 97.5 nm^2^ (5D TTX 3D recovery), and 105.7 nm^2^ (10D TTX 3D recovery; one-way ANOVA, *p* = 0.26). ***C***, Average 2D area of Bassoon puncta colocalized with synaptic partners. Means are 105.1 nm^2^ (CTRL), 86.3 nm^2^ (5D TTX 3D recovery), and 101.9 nm^2^ (10D TTX 3D recovery). Stars denote a significant one-way ANOVA with Tukey's post hoc *p* < 0.05. ***D***, Presumptive synapse density, estimated as a count of PSD-95 puncta that are colocalized with Bassoon partners, across conditions. Means are 0.41 colocalizations/μm^3^ (CTRL), 0.25 CLs/μm^3^ (5D TTX 3D recovery), and 0.18 CLs/μm^3^ (10D TTX 3D recovery). Star for significant one-way ANOVA with Tukey's post hoc both *p* < 0.05.

Most prior studies of deprivation-induced changes at cortical synapses have emphasized the role of increased excitatory postsynaptic strength (O’Brien et al., 1998; Turrigiano et al., 1998). Our measure of postsynaptic size (2D cross-sectional area of colocalized PSD-95) revealed a subtle change. Size exhibited a progressive increase as slices were silenced with TTX (Fig. 6*B*, a nonsignificant 15% increase from CTRL to 10D TTX 3D recovery; one-way ANOVA: *F* = 1.41, *p* = 0.26). The divergence between this result and consistent reports of increased postsynaptic size after acute deprivation, including in a recent study also looking at prolonged TTX in cortical organotypic culture (Wise et al., 2023), likely reflects some degree of reversibility of synaptic size during a period of recovery.

The relationship between the progressive silencing and the size of the presynaptic Bassoon puncta was more complex and nonmonotonic. Bassoon 2D area first decreased after 5D of TTX but returned to control levels following 10D of treatment and 3D of recovery, resulting in a significant modification of size with treatment (Fig. 6*C*, *F* = 5.95, *p* < 0.01). Post hoc tests of condition differences were significant for the 5D TTX condition relative to CTRL (*p* = 0.01) and to 10D TTX (*p* = 0.04), but CTRL and 10D TTX were not significantly different (*p* = 0.87). This does not accord with the results of deprivation prior to recovery, which has found matching increases in both pre- and postsynaptic sizes (Mitra et al., 2011; Wise et al., 2023). This discrepancy could reflect the effects of elevated activity during the recovery period.

Presumptive excitatory synapses were also reduced in density over increasing TTX duration (Fig. 6*C*). Synapse density, defined as the number of PSD puncta colocalized with a Bassoon presynaptic partner per unit area, dropped sharply with increasing duration of silencing. This 57% loss of excitatory synapses was significant by 10D of TTX, whereas a 42% decrease was not significant at 5D of TTX (one-way ANOVA, *F* = 5.10, post hoc testing, *p* = 0.01; *p* = 0.06 for CTRL vs 5D TTX 3D recovery, *p* = 0.01 for CTRL vs 10D TTX 3D recovery, *p* = 0.64 for 5D TTX 3D recovery vs 10D TTX 3D recovery). A comparable decrease was recently observed acutely after 5D deprivation without recovery (Wise et al., 2023). Our results may suggest that the network is unable to restore excitatory synapse density within 3 d.

**Figure 7.**
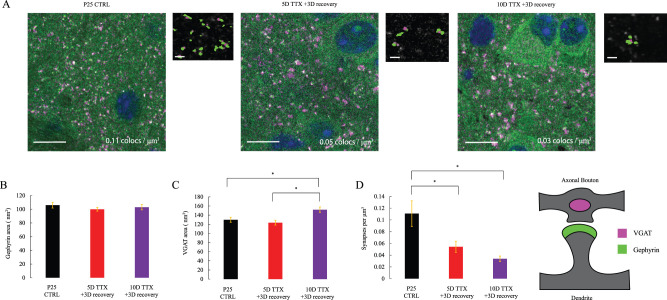
Inhibitory synapse density progressively declines with increasing duration of silencing as the presynaptic inhibitory protein marker increases in size. ***A***, Representative *z*-frames of slices, as in Figure 4, stained at the postsynapse (gephyrin, green), and presynapse (VGAT, magenta). The scale bar on the large image is 10 μm. The right panel of each duo shows a zoomed-in (100 pixel × 100 pixel, or 72 μm square) view of a single area with thresholded colocalized puncta pseudocolored and a background of the gephyrin stain in greyscale. The scale bar on the small image is 1 μm. *n* = 12 EP25 CTRL slices, 12 EP25 5D TTX 3D recovery, 14 EP25 10D TTX 3D recovery. ***B***, Average 2D cross-sectional area (in frame with brightest pixel) within a 0.72 μm × 0.72 μm × 3 μm stack of gephyrin puncta colocalized with a synaptic partner, as in Figure 5 (age-matched control, 5D TTX + 3D recovery and 10D TTX + 3D recovery). Means are 105.9 nm^2^ (CTRL), 99.9 nm^2^ (5D TTX 3D recovery), and 103.0 nm^2^ (10D TTX 3D recovery). One-way ANOVA *p* = 0.54. ***C***, Average 2D area of VGAT puncta colocalized with a synaptic partner. Means are 129.9 nm^2^ (CTRL), 123.5 nm^2^ (5D TTX 3D recovery), and 151.9 nm^2^ (10D TTX 3D recovery). Stars represent one-way ANOVA and post hoc Tukey's with *p* < 0.05. ***D***, Gephyrin colocalized puncta density. Means are 0.11 colocalizations/μm^3^ (CTRL), 0.05 CLs/μm^3^ (5D TTX 3D recovery), and 0.03 CLs/μm^3^ (10D TTX 3D recovery). Stars represent one-way ANOVA and post hoc Tukey's, also with *p* < 0.05.

Network activity depends not only on intrinsic excitability and excitatory synaptic strength and number but also critically on the strength and number of inhibitory synapses. Inhibitory synapse density was previously shown to decrease with silencing (Chattopadhyaya et al., 2004), with concomitant synaptic weakening (Kilman et al., 2002). We labeled inhibitory presynaptic terminals with VGAT and postsynapses with gephyrin. Previously observed reductions in size were not observed following deprivation with a period of recovery. Here, postsynaptic gephyrin puncta were not significantly altered in size (Fig. 7*B*, one-way ANOVA *F* = 0.62, *p* = 0.54), while presynaptic VGAT puncta were larger following 10D silencing (Fig. 7*C*, one-way ANOVA: *F* = 7.42, *p* < 0.01; *p* = 0.71 for CTRL vs 5D TTX 3D recovery, *p* = 0.02 for CTRL vs 10D TTX 3D recovery, *p* < 0.01 for 5D TTX 3D recovery vs 10D TTX 3D recovery). These modest changes in inhibitory synapse size were dwarfed by a profound and progressive reduction in inhibitory synapse number (Fig. 7*D*, 67% decrease from CTRL to 10D TTX, 51% decrease at 5D TTX), which was highly significant as a one-way ANOVA (*F* = 8.94, *p* < 0.01) and represented a significant shift with deprivation from age-matched CTRL (*p* = 0.02 vs 5D TTX 3D recovery; *p* < 0.01 vs 10D TTX 3D recovery) although not between the two deprived conditions (*p* = 0.53). These results indicate that the loss of inhibitory synapses is greater than the loss of excitatory synapses, suggesting a reduction in the E/I balance likely to favor the emergence of epileptiform activity and circuit hyperexcitability (Nelson and Valakh, 2015).

### No cell death induced by treatment with TTX

Organotypic slices cultured in the presence of TTX exhibited an obvious visual thinning of the tissue that, if shown to indicate significant cell loss, might account for some or all of the decrease in synapse count (Dingledine et al., 2014). We counted nuclei stained with a DAPI stain from layer five of the primary somatosensory cortex at 10× on a confocal microscope (Figure 1-1), over a *z*-stack containing the whole thickness of the same slices used for synapse imaging, on the three conditions of increasing silencing duration assayed prior. There was no difference across conditions in cell count (one-way ANOVA, *F* = 0.61, *p* = 0.55). This measure offers no insight into whether there is a change in the relative mixture of different cell types (such as a loss of neurons accompanied by an increase in astrocyte density) because DAPI binds to DNA in the nucleus of all cells. Nevertheless, we can be confident that massive cell death did not occur during our deprivation experiments.

## Discussion

Prior studies of the response of central circuits to activity deprivation have mostly focused on brief, rapidly reversible homeostatic changes that normally allow circuits to maintain stable firing rates. Here, we have focused instead on the progressive and slowly reversing compensatory plasticity that eventually leads to hypersynchronous seizure-like activity. The observed plasticity shares many features in common with the seizure disorder that develops in vivo during chronic activity blockade (Galvan et al., 2000; Lee et al., 2008). First, as in vivo, the observed network activity became progressively stronger during longer periods of deprivation. Second, as in vivo, the effect of deprivation was strongest when begun prior to the end of the second postnatal week, a period corresponding to a period of heightened (Silverstein and Jensen, 2007; Aaberg et al., 2017) risk of developing seizures in human patients. Third, the activity is slow to recover, showing incomplete recovery after 3 d and only showing full recovery in gross network activity levels after a period equal to that of the deprivation (10 d). Limitations of the ability to maintain healthy slice cultures for very long periods restricted our ability to look at longer periods of deprivation and longer periods of recovery. In vivo, seizures take 10–14 d of disruption to develop but then do not recover. However, this may involve many brain regions and perhaps require changes set in motion once seizures begin. It may be that while our disturbed networks can produce nominally normal firing at 10 d of recovery, they may still be more vulnerable to insult or occasionally produce epileptiform bursts consistent with occasional seizures (roughly every 3.1 h in Galvan et al., 2000). Although we could not rule out such lasting effects of seizures in vivo, we did rule out the idea that persistent hyperactivity was due to Hebbian plasticity induced by epileptiform rebound bursting. Blocking NMDA receptors during this period did not lead to more rapid recovery, arguing against the idea that epileptiform activity was “locked in” by Hebbian LTP during synchronous network activity.

### Activity deprivation, homeostatic mechanisms, and seizure disorders

The effects of activity deprivation may have broader application to more common forms of seizure disorders. Epilepsy can begin following a stroke or injury (Curia et al., 2016), which can trigger additional waves of cell death in the cortex, especially in young animals (Bittigau et al., 2003). Much is still unknown about how seizures result from trauma. Interestingly, neuronal presynaptic terminals become silent during excitotoxic insults, perhaps in a neuroprotective effort to avoid further damage, resulting in a period of abnormally reduced neuronal firing (Hogins et al., 2011). This progression from hypoactivity to hyperactivity has been shown in a controlled cortical impact model of TBI (Ping and Jin, 2016). Cortical deafferentation, another deprivation paradigm modeling TBI-induced epilepsy (Fröhlich et al., 2008), has been linked to homeostatic readjustment previously, causing increased excitatory neuron intrinsic excitability and input resistance (Avramescu and Timofeev, 2008) as well as excitatory synapse scaling (González et al., 2015). Stimulation of the cortex can provide relief from certain consequences of TBI, such as tinnitus, acquired epilepsy, and neuropathic pain (Chai et al., 2019). This suggests that the maladaptive compensatory plasticity observed in vivo and in vitro may have broader relevance to other pathological conditions giving rise to seizure disorders.

### Mechanisms of prolonged versus short-term homeostatic compensation

Prior mechanistic studies of the homeostatic response to activity blockade have emphasized the importance of three coordinated mechanisms: (1) a strengthening of excitatory synapses through postsynaptic upscaling, presynaptic enhanced release, or both; (2) a weakening of inhibitory synapses, also potentially occurring pre- and postsynaptically; and (3) an increase in intrinsic excitability occurring either globally, or selectively involving the sensitivity, size, and placement of the axon initial segment (Turrigiano and Nelson, 2004; Wefelmeyer et al., 2016; Tien and Kerschensteiner, 2018).

Surprisingly, we found limited support for the idea that persistent network hyperexcitability involved a lasting increase in the number or strength of excitatory synapses. Specifically, we found that excitatory synapse number decreased by 57% during the course of 10 d of deprivation followed by 3 d of recovery. A more intermediate value after 5 d of deprivation and 3 d of recovery was not significant. Prior studies of activity deprivation have attributed a similar progressive decrease in excitatory synapse density (Minerbi et al., 2009) to a failure to stabilize transient synapses (De Roo et al., 2008a,b). Loss of excitatory synapses, while operating in the opposite direction expected to address an activity deficit, could still contribute to synchronicity if fewer synapses are unable to meet the demanding tuning requirements of a balanced circuit state (Atallah and Scanziani, 2009; Treviño, 2016).

There was no structural evidence of a lasting significant increase in synaptic size. A recent study in neocortical slice culture demonstrated that 5 d of deprivation without an additional recovery period produced increases in synapse size evident both pre- and postsynaptically and apparent in both super-resolution light microscopy and ultrastructurally (Wise et al., 2023). The difference between the present results and those obtained in the Wise study and in multiple other studies of deprivation-induced homeostatic plasticity where postsynaptic increases in excitatory synapse size are consistent and presynaptic increases are sometimes observed (O’Brien et al., 1998; Turrigiano et al., 1998; Murthy et al., 2001; Mitra et al., 2011; Barnes et al., 2017; Hobbiss et al., 2018) suggests that this prominent feature of synaptic change may readjust rapidly as activity changes and that deprivation-induced increases in synaptic size were likely reversed by elevated activity during the 3 d recovery period. Indeed, when TTX is applied to organotypic hippocampal cultures for a much shorter time (48 h), synaptic scaling results in increased mEPSC amplitude acutely but presents a complete recovery when slices recover in media for 1 week (Koch et al., 2010). Because we did not obtain physiological measurements of spontaneous or evoked synaptic transmission, we cannot rule out the possibility of a lasting increase in the probability of presynaptic release. However, in all the prior studies just mentioned, increased release was accompanied by increased presynaptic size, which was not seen here.

We furthermore did not observe evidence of a lasting decrease in the size of individual inhibitory synapses as reported previously in dissociated culture (Kilman et al., 2002; Swanwick et al., 2006). In fact, after 10 d of activity blockade, followed by 3 d recovery, puncta of colocalized presynaptic VGAT showed a modest, but significant increase. Elevated extracellular potassium, which depolarizes cells and creates a hyperexcitable regime, is known to enlarge inhibitory synapses over 48 h (Rannals and Kapur, 2011). During the overshoot period in our model, a similar hyperexcitability is induced. The increase we see in inhibitory presynapse size, therefore, could reflect an indirect transient change to address the elevated activity of the recovery period and not a direct response to deprivation. However, because we focused most of our efforts on identifying persistent circuit alterations after a period of recovery, we cannot rule out the possibility that the failure to observe significant increases in excitatory and inhibitory postsynaptic size was already present prior to recovery. Pre- and postsynaptic changes in synaptic size may occur with independent kinetics, and these kinetics could further differ for excitatory and inhibitory synapses, especially since the two types of synapses differ both in their postsynaptic components and in the presynaptic neuron type. Mapping these kinetics would require a more detailed parametric study of deprivation and recovery times than was completed here.

In contrast to the small increase in presynaptic inhibitory size, we also saw persistent and dramatic changes in the number of presumptive inhibitory synapses. These synapses were reduced to half of their initial density by 5 d and to a third of their initial density after 10 d of deprivation, and in both cases, these reductions were persistent since they were apparent after a 3 d recovery period. Inhibitory synapse development has long been known to depend bidirectionally on circuit activity. Elevated activity increases the number of inhibitory synapses (Marty et al., 2000) as well as their size (Rannals and Kapur, 2011), and reduced activity decreases inhibitory synapse density (Chattopadhyaya et al., 2004) and/or size (Kilman et al., 2002). Epilepsy has also been linked to inhibitory synapse loss in humans (Calcagnotto et al., 2005; Alonso-Nanclares et al., 2011). Overall reduced inhibition likely contributed to the persistent epileptiform activity evident here after prolonged deprivation in slice culture.

Although both excitatory and inhibitory putative synapse density decreased, the loss was greater for inhibitory synapses (57% for excitatory synapses vs 67% for inhibitory synapses). The balance between excitation and inhibition in neural circuits has been long understood as a functional requirement of healthy activity propagation (Zhou and Yu, 2018; Sohal and Rubenstein, 2019; Chen et al., 2022). This shift in propagation equilibrium is likely a contributor to the hypersynchronous circuit resulting from activity deprivation.

### Intrinsic excitability

In addition to a lasting reduction in inhibition, we also observed a lasting increase in intrinsic excitability. Layer 5 pyramidal cells exhibited a roughly twofold increase in input resistance and an elevated FI-slope after 10 d of deprivation and showed an intermediate, but nonsignificant level of firing after 5 d of TTX. Attempts to quantify the impact of deprivation prior to recovery were difficult because nearly all silenced cells exhibited depolarization block curtailing bursting behavior (data not shown). Many cells after recovery exhibit a standard firing curve, suggesting incomplete but substantial recovery of normal firing properties. Plasticity of intrinsic excitability in a homeostatic context was described alongside the early work on synaptic scaling (Desai et al., 1999), with excitability rising to produce greater firing rates following deprivation. Modeling studies have suggested that homeostatic intrinsic plasticity might contribute to seizures following brain injury and associated silencing (Houweling et al., 2005). Intrinsic excitability-based homeostatic plasticity is thought to be developmentally regulated (Wen and Turrigiano, 2021), which might explain in part how activity deprivation is so devastating during early postnatal development. In any case, although it is not surprising that neurons become more excitable after deprivation, we were surprised to find that these changes could persist after 3 d of recovery. These biophysical properties of the cell are very important to network function, and the persistent shifts we observed in input resistance and firing output in pyramidal excitatory neurons push the cortical network toward persistent hyperexcitability.

## Conclusion

In conclusion, during protracted activity deprivation, maladaptive compensatory changes in cortical circuits continue to accrue. Much as occurs with in vivo activity blockade, these changes are developmentally regulated and progressive and do not require Hebbian plasticity. Unlike in vivo, hyperactivity in cortical slice culture eventually recovers although this process takes many days. Mechanistically, we found that both inhibitory and excitatory synapses were lost, with a greater loss of inhibition, leading to an imbalanced circuit. In addition, changes in cellular excitability were surprisingly persistent and so likely also contributed to circuit hyperexcitability. Operationally, the slice culture preparation studied here may provide a useful testbed for future studies of pathophysiological mechanisms and potential treatments for seizure secondary to activity deprivation.

## Synthesis

Reviewing Editor: Niraj Desai, National Institute of Neurological Disorders and Stroke Decisions are customarily a result of the Reviewing Editor and the peer reviewers coming together and discussing their recommendations until a consensus is reached. When revisions are invited, a fact-based synthesis statement explaining their decision and outlining what is needed to prepare a revision will be listed below. The following reviewer(s) agreed to reveal their identity: Graham Diering, Mathieu Letellier.

Your manuscript has now been read by two reviewers and by me. We were all impressed by the work, and we all remarked on the logic and clarity of the presentation. This manuscript was simply a pleasure to read. Good job and thank you.

One reviewer (see comments below) had some suggestions for improving a few parts. These suggestions are relatively minor: they do not necessitate additional experiments (unless you think otherwise) and should not require substantial new work.

I look forward to reading your resubmission soon.

REVIEWER #1

Here the authors examine synaptic and circuit activity changes in organotypic cortical slices following prolonged 5-10 activity silencing using TTX and recovery following TTX removal. The authors demonstrate that circuit changes following network silencing lead to long lasting changes in synapse number and properties, as well as changes in neuronal excitability measures. These changes take days to recover following a 5day silencing paradigm, but do not fully recover following a 10-day silencing. The latter point is consistent with prolonged activity changes contributing to epileptogenesis. Initially following the activity silencing, cortical networks exhibit hyper synchrony and hyperactivity. These changes are accompanied by a surprising decrease in excitatory synapse density, seemingly at odds with network hyperactivity. However, the authors also demonstrate an even larger decrease in inhibitory synapse density, and large increase in excitability measures, that readily explain network changes. This type of prolonged activity manipulation and recovery paradigm is quite novel and not well represented in the literature. Thus, this work provides new mechanistic insights into network adaptations and synapse homeostasis. The use of organotypic cultures for this ideal for this project and a real advantage.

The conclusions drawn by the authors are well supported by strong data. The manuscript is excellently prepared, the discussion is stimulating and appropriately addresses considerations for technical items or interpretation of mechanisms. The figures are clear, reporting of methods and statistics is excellent. Overall, the entire manuscript is very strong. Thre are no concerns to be addressed.

REVIEWER #2

In this article, the authors delve into the cellular and physiological mechanisms underpinning rebound activity following an extended period of activity blockade, a phenomenon that may contribute to the onset of seizures in the forebrain. Using calcium imaging, synaptic staining, and whole-cell patch-clamp recordings in cortical organotypic slices, the authors observed that the rebound activity could persist for several days following the reinstatement of activity but gradually returned to baseline levels after more extended recovery periods. This rebound activity was age-dependent and correlates with several key factors, including heightened intrinsic excitability and input resistance, an altered balance between excitation and inhibition, and a reduction in the density of both excitatory and inhibitory synapses. Collectively, these findings shed light on a compelling aspect of homeostatic plasticity responding to chronic inactivity, highlighting its potential to drive maladaptive circuit alterations.

The methodology employed in this article are sound and well-suited to the research objectives. Additionally, the article is logically written. Nevertheless, there are some concerns that warrant the authors' attention and clarification before publication:

1) P9, the authors assert that "A three-day period of recovery in normal media results in an incomplete recovery that is correspondingly less restorative when the deprivation is more prolonged." However, it's worth noting that in the experiments shown in Figure 1, the observed disparity between 5D TTX and 10D TTX outcomes might not solely be attributed to the duration of the blockade. There could also be an age-related factor at play, as indicated by the authors themselves and demonstrated in Figure 3. In fact, when considering measures of synchrony and stereotypy, the impact of 5D TTX starting at EP12 (as shown in Figure 3) appears similar to the impact of 10D TTX starting at EP12 (as displayed in Figure 1). This suggests that the influence of TTX treatment may be more contingent on the developmental stage than the sheer duration of treatment. Consequently, the authors should consider revising their interpretation or, alternatively, conducting additional experiments involving a 10D TTX treatment starting at EP17 instead of EP12 to further elucidate these differences.

2) It is not clear why the authors did not quantify the upstate duration in experiments shown in Figure 2, 3 and 4 as they did for Figure 1. They should clarify this. Also, on the average trace shown in Figure 1A, the upstate duration after recovery seems not representative of the measure shown in Figure 1H. The authors should provide an explanation for this.

3) The authors should explain in which region of the slices they performed their synaptic analysis, as synapse density and synapse type may vary across cortical layers.

4) It would have been nice to have the immunostaining data after the TTX treatment and before the recovery period in order to conclude on whether the effect on synapse size and number results from activity blockade or from the recovery period. In particular, regarding the divergent effect on pre and postsynaptic size, the authors acknowledge P14: "This does not accord with the results of deprivation prior to recovery, which have found matching increases in both pre- and postsynaptic size (Mitra et al., 2011; Wise, Escobedo-Lozoya et al., 2023). This discrepancy could reflect the effects of elevated activity during the recovery period." I understand that the effect of prolonged activity in synapse density and size have been explored in many previous studies but this was not necessarily performed in the same experimental paradigm and I think this information is missing here to better isolate the effect of the recovery period. Also, could the authors elaborate on how the activity rebound could lead to a divergent effect on pre and postsynatpic sizes?

5) For some of the data, the statistical analysis is not described (e.g., Figure 5B; Figure 6B-D, etc.). The authors should provide the information for every panels in each figure.

## Author Response

Dear Dr. Desai and Reviewers, We greatly appreciate your careful reading and suggestions for the improvement of our manuscript. In response to these comments we have made various changes to the manuscript, adding several figure panels and adjustments to the text.

Synthesis Statement:

Thank you for the positive feedback.

Reviewer #1 had no suggestions for improvements.

Reviewer #2 1) P9, the authors assert that "A three-day period of recovery in normal media results in an incomplete recovery that is correspondingly less restorative when the deprivation is more prolonged." However, it's worth noting that in the experiments shown in Figure 1, the observed disparity between 5D TTX and 10D TTX outcomes might not solely be attributed to the duration of the blockade. There could also be an age-related factor at play, as indicated by the authors themselves and demonstrated in Figure 3. In fact, when considering measures of synchrony and stereotypy, the impact of 5D TTX starting at EP12 (as shown in Figure 3) appears similar to the impact of 10D TTX starting at EP12 (as displayed in Figure 1). This suggests that the influence of TTX treatment may be more contingent on the developmental stage than the sheer duration of treatment. Consequently, the authors should consider revising their interpretation or, alternatively, conducting additional experiments involving a 10D TTX treatment starting at EP17 instead of EP12 to further elucidate these differences.

We agree that changes in excitability likely result from a combination of deprivation time and developmental period when the silencing began. While we are past the opportunity to perform additional experimentation of this type, we did already perform additional experimental time points that we did not report in the submitted manuscript. We've decided to add these additional experiments to our text but think their overall importance falls short of adding an entire figure.

We did not measure the response to 10D TTX begun at EP17, but we did ask whether the degree of recovery depended on the developmental stage at which 5D TTX was begun by also measuring the response to 5D TTX, 3D recovery begun at EP12, rather than EP17. This produced an intermediate activity state between 10D TTX and control samples, similar to 5D TTX starting at P17 (and displaying similar values for synchrony and stereotypy). We added the following text to the segment titled "Calcium transients demonstrate a progressive loss of activity homeostasis with prolonged deprivation": "It is possible that, since our recovery analysis compared cultures that had 10D of TTX beginning at EP12 to cultures that had 5D of TTX beginning at EP17, the earlier initiation of prolonged silencing was a better explanation than the increased duration for why the cultures remain persistently disrupted. However, we also tested recovery after 5D TTX when deprivation began at EP12. While the initial rebound hyperactivity from 5D TTX at this earlier age is somewhat more severe (n = 17 EP17, n = 14 EP22; synchrony mean 0.94 at EP17, 0.87 at EP22; stereotypy mean 0.66 at EP17, 0.44 at EP22), there was similar recovery after 3D of recovery at the two ages. EP20 3D recovery showed 0.74 mean synchrony, compared to EP25 3D recovery at 0.78 mean synchrony (n = 10 EP20, n = 14 P25; two-tailed T-test p = 0.40); stereotypy mean 0.40 for EP20, mean 0.33 for EP25 (two-tailed T-test p = 0.22). Repeating the 2-way ANOVA to compare these effects to 10D TTX and to condition-matched CTRL, resulted in similar conclusions to those obtained from the measurements made at EP25. With regards to synchrony, 5D TTX starting EP12 was still statistically significantly different from CTRL (Tukey's p < 0.01), but the somewhat higher value was intermediate to but now not significantly different from 10D TTX 3D recovery starting at EP12 (p = 0.41). Stereotypy, as above, reveals a full recovery at 3D following 5D TTX starting EP12 (p = 0.85 vs. CTRL), but is now not significantly more recovered than 10D TTX 3D recovery starting at EP12 (p = 0.14). We conclude that 5D TTX at this earlier time point is insufficient to yield lasting hyperactivity, and still produces an intermediate state to 10D TTX starting at the same time." 2) It is not clear why the authors did not quantify the upstate duration in experiments shown in Figure 2, 3 and 4 as they did for Figure 1. They should clarify this. Also, on the average trace shown in Figure 1A, the upstate duration after recovery seems not representative of the measure shown in Figure 1H. The authors should provide an explanation for this.

We have added descriptions of the effect of our various treatments on up state duration to the text in the results section dealing with Figures 2-4. Specifically we added: "... and duration (p = 0.81)..." to p. 9 "Duration of upstate events remains briefer following deprivation, no matter what age silencing begins (p < 0.01 at all ages, Figure 3F)" to p. 10 "... duration p = 0.99)..." and "... though duration of up states is somewhat statistically closer to baseline following recovery in APV than without APV due to variance (p = 0.19)" to p. 11 We replaced the example trace for Figure 1A with a sample that is closer to the average for all measures, including upstate duration.

3) The authors should explain in which region of the slices they performed their synaptic analysis, as synapse density and synapse type may vary across cortical layers.

We have added the statement "Imaging was confined to layer 5 of primary somatosensory cortex" to the Synaptic Imaging segment of the methods section.

4) It would have been nice to have the immunostaining data after the TTX treatment and before the recovery period in order to conclude on whether the effect on synapse size and number results from activity blockade or from the recovery period. In particular, regarding the divergent effect on pre and postsynaptic size, the authors acknowledge P14: "This does not accord with the results of deprivation prior to recovery, which have found matching increases in both pre- and postsynaptic size (Mitra et al., 2011; Wise, Escobedo-Lozoya et al., 2023). This discrepancy could reflect the effects of elevated activity during the recovery period." I understand that the effect of prolonged activity in synapse density and size have been explored in many previous studies but this was not necessarily performed in the same experimental paradigm and I think this information is missing here to better isolate the effect of the recovery period. Also, could the authors elaborate on how the activity rebound could lead to a divergent effect on pre and postsynaptic sizes? Although we agree with the reviewer that adding the two additional time points to the synaptic puncta quantification would be helpful, we do not currently have the resources to repeat these experiments at additional timepoints and so prefer to temper our conclusions rather than further delay publication.

We've added a recognition of this shortfall in the discussion section (p. 18): "However, because we focused most of our efforts on identifying persistent circuit alterations after a period of recovery, we cannot rule out the possibility that the failure to observe significant increases in excitatory and inhibitory postsynaptic size was already present prior to recovery." The reviewer also asks us to elaborate on how activity rebound can have divergent effects on pre- and post-synaptic size. We have added the following to the discussion section (p. 18): "Pre- and postsynaptic changes in synaptic size may occur with independent kinetics, and these kinetics could further differ for excitatory and inhibitory synapses, especially since the two types of synapses differ both in their postsynaptic components, and in the presynaptic neuron type. Mapping these kinetics would require a more detailed parametric study of deprivation and recovery times than was completed here." 5) For some of the data, the statistical analysis is not described (e.g., Figure 5B; Figure 6B-D, etc.). The authors should provide the information for every panels in each figure.

The statistical description for these analyses was always included in the text, but was not always included in the figure legend itself. We have updated the figure legends to include a summary of this information as well. We also now explicitly explain that graphical indications of significance have a uniform meaning across Figures 1-4.

We hope that with these changes the manuscript will now be suitable for publication. Thanks again for your help improving it.

Sincerely, Derek Wise and Sacha Nelson, on behalf of the rest of the authors
