# Supplementary figures and images for: Progressive Circuit Hyperexcitability in Mouse Neocortical Slice Cultures with Increasing Duration of Activity Silencing
Source: eNeuro. 2024 May 2;11(5):ENEURO.0362-23.2024. doi: 10.1523/ENEURO.0362-23.2024 (PMC11079856; doi:10.1523/ENEURO.0362-23.2024)

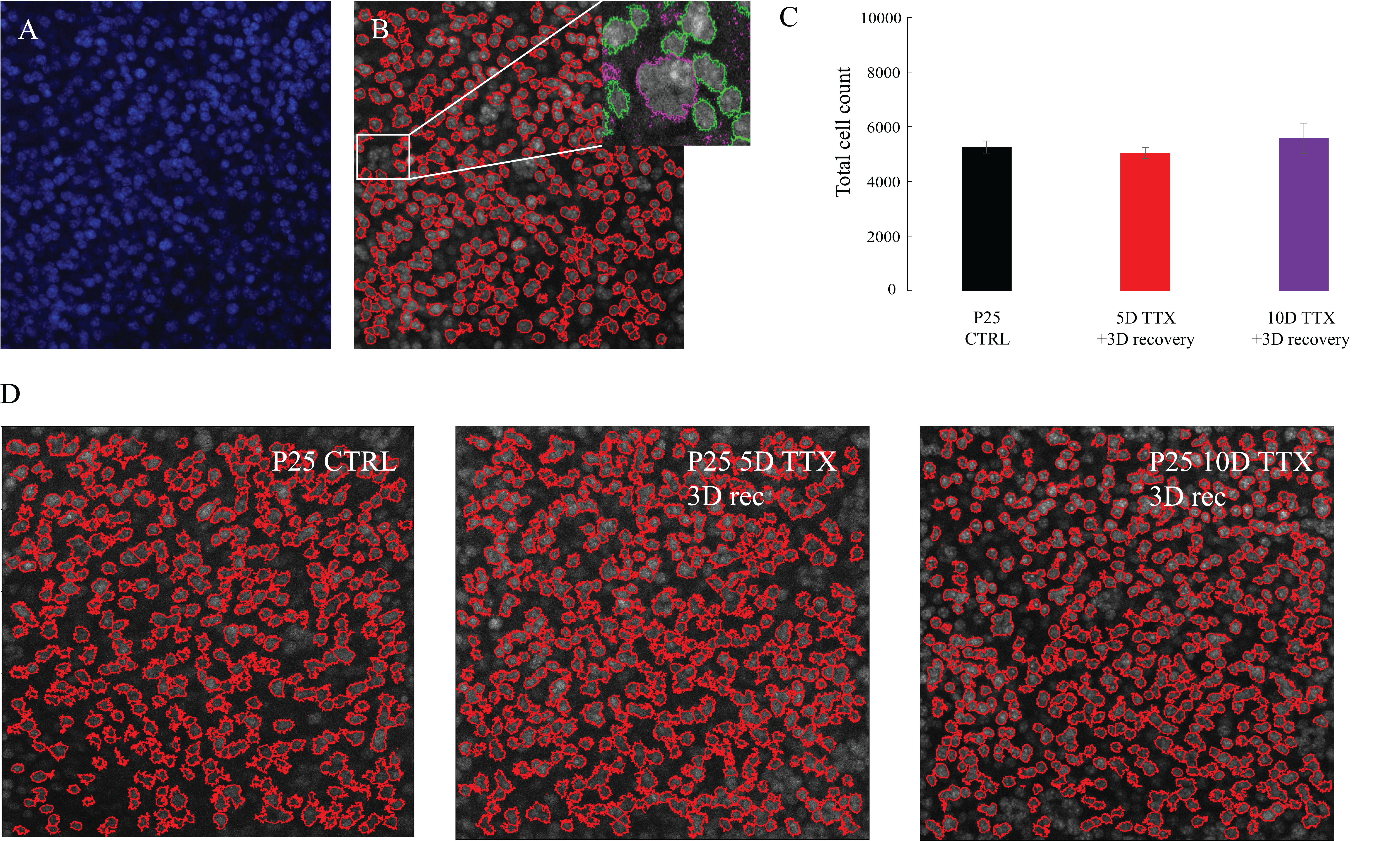

Supplement: Figure 1-1 — No significant cell death occurs with prolonged tetrodotoxin exposure. A. Example 10x image from Zeiss Airyscan of DAPI-stained nuclei (blue) in layer 5 of somatosensory cortex in 461 x 461 μm area. Full z extent was imaged for each stack, so the total volume differed in for each sample. B. CellProfiler calls (red outline) of good nuclei with inset showing examples of rejected blurred nuclei out of focus (purple outlines) and accepted crisp outlines of counted nuclei (green outlines). C. Bar graph of total nuclei count in our three conditions, from the same slices used in synaptic imaging analysis. 1-way ANOVA and posthoc Tukey’s both p > 0.05. D. Examples of accepted cell density (red outlines) from all three conditions. Download Figure 1-1, TIF file. [file eneuro-11-ENEURO.0362-23.2024-s001.tif]
